# Spontaneous rupture of an unscarred uterus in a woman at 37 weeks of pregnancy with abdominal pain: a case report

**DOI:** 10.1016/j.xagr.2022.100082

**Published:** 2022-08-06

**Authors:** Sophie Locher, Mohamed A. Jellouli, Jérome Mathis, Duc E. Ha

**Affiliations:** Frauenklinik, Centre Hospitalier Bienne, Bienne, Switzerland.

**Keywords:** Unscarred uterus, Uterine rupture, Pregnancy, Case report

## Abstract

A 34-year-old gravida 2, para 1 woman at 37+4 weeks of pregnancy presented with abdominal pain. She had no medical history. Complete examination was unremarkable. After hours of monitoring, the patient abruptly deteriorated. An emergency cesarean delivery revealed a ruptured uterus with significant issues. Cautious monitoring is essential for such patients with atypical pain.

## Case report

A 34-year-old gravida 2, para 1 woman at 37+4 weeks of pregnancy presented to the hospital with unclear persistent abdominopelvic pain for 10 hours, radiating from the left side to the whole abdomen. The patient had no medical, surgical, or gynecologic history. Her obstetrical history revealed a vaginal delivery 2 years ago. The current pregnancy was without any relevant finding.

At admission, the patient was apyretic, normocardial, and normotensive. The abdominal palpation was globally painful, without contracture or contractions.

Cardiotocography showed a fetal heart rate that was normocardial and normoreactive, and some unregular uterine contractions. The laboratory and urinary values were nonspecific.

Sonographic examination found no evidence of placental abruption or uterine rupture. The fetus was in a cephalic position, with a good heart frequency and vitality, and enough liquid. The rest of the maternal abdomen examination showed a small right pyelocaliceal dilatation (1.5 × 1.8 cm) and a suspected small free fluid in the Morison pouch. Faced with the large discordance between acute pain and absence of obvious abnormalities during exploration, we decided to proceed with close observation of the patient in the delivery room (heart frequency, blood pressure, fetal heart monitoring), in association to an efficient analgesic treatment.

Four hours later, while standing up, the patient suddenly exhibited palor, an excessive decrease in blood pressure, and exacerbated abdominal pain. Fetal monitoring showed an abrupt severe fetal heart rate deceleration. An emergency cesarean delivery was then performed under general anesthesia.

On accessing the abdominal cavity, we found a hemoperitoneum of 200 mL. After delivering a 2655-g neonate by classical isthmic transverse uterotomy, we identified a longitudinal tear in the left lateral wall of the uterus of approximately 7 cm, involving also the left parametrium (Figure). The rupture was sutured by separate X-stitches with absorbable sutures. Careful examination of the rest of the cavity revealed no other signs of rupture. Notably, we found a closed adhesion of the small intestine to the left sacrouterine ligament, which was respected. In total, the patient lost 1500 mL of blood and was transfused with 2 erythrocytes pellets.

**Figure:**
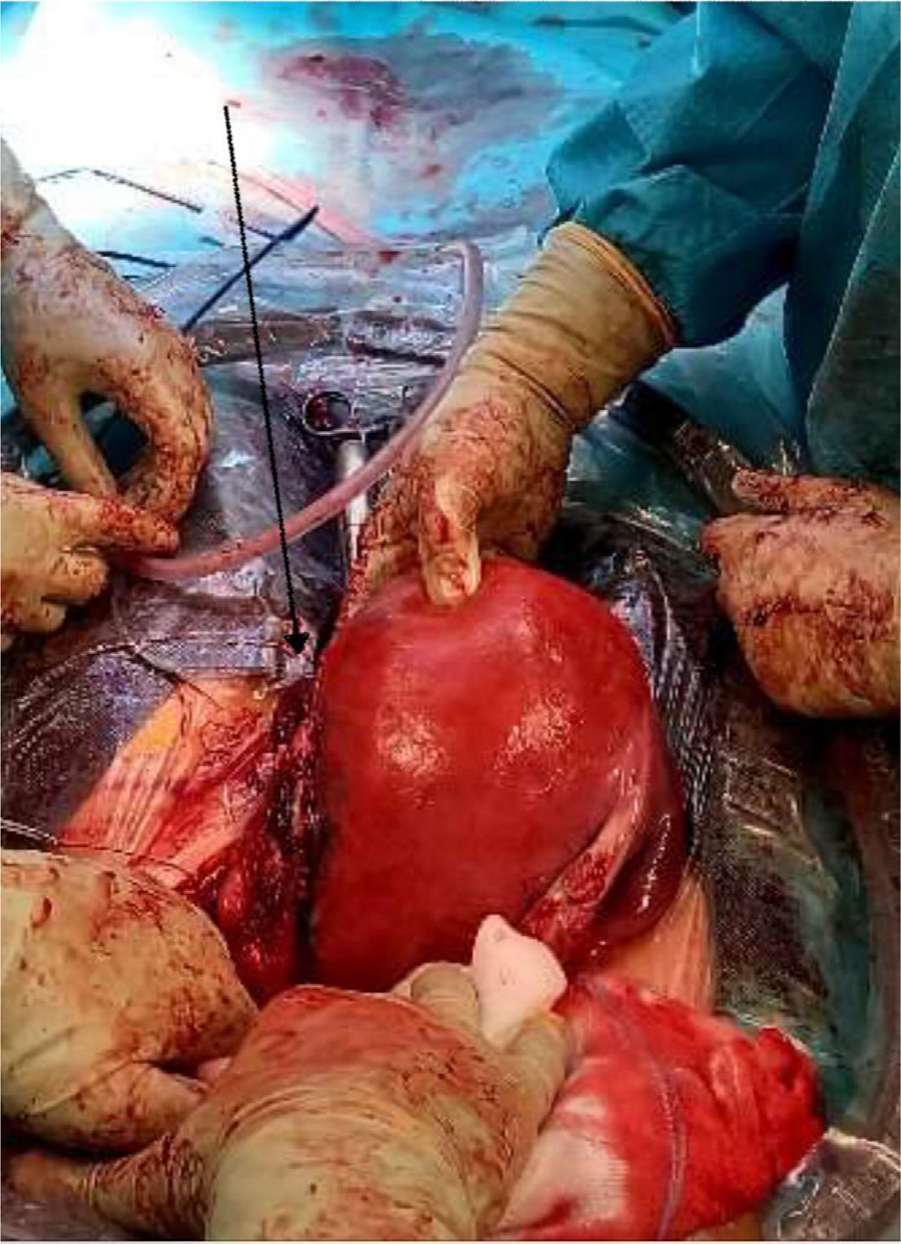
Left lateral uterine rupture

Locher. Case report of a spontaneous rupture of an unscarred uterus. Am J Obstet Gynecol Glob Rep 2022.

The postoperative outcome was favorable, and the mother and infant were able to leave the maternity ward after 6 days. Analysis of the placenta did not reveal any pathology.

Spontaneous uterine rupture is a rare life-threatening obstetrical complication. It usually occurs during labor after a previous cesarean delivery or any previous uterine procedures such as myomectomy or any other surgical intervention. There are also other risk factors, such as shortened interdelivery interval, pregnancy of >40 weeks’ gestation, neonatal birthweight >4000 g, or labor induction with prostaglandins.

We have found very few publications in the literature about cases of uterine rupture in pregnant women reaching term with no history of uterus scarring and without clear labor contractions. Parant^1^ noted that the diagnosis of uterine rupture relies on cardiotocographic abnormalities and unexplained and unusual abdominal pain, which was also observed in our patient's case. Retrospectively, the only potential risk factor of this patient was suspected chronic inflammation indicated by a closed adhesion of the small intestine to the left sacro uterine ligament, which was not reported by the patient.

A lateral injury of unscarred uterus is associated with a high risk of maternal and fetal complications after rupture.^2^ This can be linked to the unusual presentation of these cases and the resultant delayed diagnosis. We thankfully did not observe severe maternal or fetal complications, which was likely attributable to close observation of the patient. Uterine rupture in unscarred uterus is rare but not exceptional. Close observation for any atypical pain during pregnancy is essential to avoid potential severe complications, especially if the indication for a cesarean delivery is not yet clear.
